# Extracellular Vesicles in Liver Transplantation: Current Evidence and Future Challenges

**DOI:** 10.3390/ijms241713547

**Published:** 2023-08-31

**Authors:** Nicola De Stefano, Alberto Calleri, Angelo Corso Faini, Victor Navarro-Tableros, Silvia Martini, Silvia Deaglio, Damiano Patrono, Renato Romagnoli

**Affiliations:** 1 General Surgery 2U-Liver Transplant Unit, Department of Surgical Sciences, Azienda Ospedaliero Universitaria Città Della Salute e Della Scienza Di Torino, University of Turin, Corso Bramante 88-90, 10126 Turin, Italy; n.destefano@unito.it (N.D.S.); renato.romagnoli@unito.it (R.R.); 2 Gastrohepatology Unit, Azienda Ospedaliero Universitaria Città Della Salute e Della Scienza Di Torino, University of Turin, 10126 Turin, Italy; alberto.calleri.md@gmail.com (A.C.); smartini@cittadellasalute.to.it (S.M.); 3 Immunogenetics and Transplant Biology Unit, Azienda Ospedaliero Universitaria Città Della Salute e Della Scienza Di Torino, University of Turin, 10126 Turin, Italy; angelocorso.faini@unito.it (A.C.F.); silvia.deaglio@unito.it (S.D.); 4 2i3T, Società Per La Gestione Dell’incubatore Di Imprese e Per Il Trasferimento Tecnologico, University of Turin, 10126 Turin, Italy; victor.navarro@2i3t.it

**Keywords:** extracellular vesicles, liver transplantation, liquid biopsy, hepatocellular carcinoma, rejection, machine perfusion, stem cells, ischemia–reperfusion injury

## Abstract

Extracellular vesicles (EVs) are emerging as a promising field of research in liver disease. EVs are small, membrane-bound vesicles that contain various bioactive molecules, such as proteins, lipids, and nucleic acids and are involved in intercellular communication. They have been implicated in numerous physiological and pathological processes, including immune modulation and tissue repair, which make their use appealing in liver transplantation (LT). This review summarizes the current state of knowledge regarding the role of EVs in LT, including their potential use as biomarkers and therapeutic agents and their role in graft rejection. By providing a comprehensive insight into this emerging topic, this research lays the groundwork for the potential application of EVs in LT.

## 1. Introduction

Liver transplantation (LT) is a life-saving procedure for patients with end-stage liver disease, liver cancer, or acute liver failure. Despite significant advances in the field, the success of LT is limited by several factors, including organ shortage, and the consequent push to expand the donor pool by utilizing extended-criteria donors (ECD), and the need for lifelong immunosuppression with its side effects [1]. Machine perfusion (MP) supports graft metabolism by continuously providing oxygen and nutrients ex vivo [2]. MP devices were developed during the pioneering era of solid organ transplantation, but they were eventually replaced by static cold storage due to its cost-effectiveness and ease of use. However, the suboptimal outcomes observed with ECD grafts have rekindled interest in MP, and various techniques have been implemented in clinical practice, each with distinct principles and mechanisms of protection [2]. Currently, MP has been shown to represent a promising strategy to mitigate ischemia–reperfusion injury (IRI) and improve graft LT outcomes [2,3,4]. However, many challenges and unmet needs remain, requiring further investigation and optimization. For instance, there is a pressing need for alternative biomarkers and diagnostic tools to monitor immunosuppression, define tumor biology, and refine graft viability assessment, as well as strategies to improve organ quality or treat pre-existing disease, with the ultimate aims of maximizing access to LT and organ utilization, refining organ allocation, tailoring immunosuppression, and improving LT outcomes [5,6,7].

In recent years, the study of extracellular vesicles (EVs) has progressed remarkably thanks to advancements in isolation and characterization techniques, as well as a better appreciation of their role in intercellular communication. EVs are nanosized vesicles enclosed by a bilayer membrane that derive from cells through two distinct biogenesis pathways. Microvesicles, also known as microparticles, are formed through the outward budding and fission of the plasma membrane and typically range in size from 100–1000 nm. In contrast, exosomes originate from the endosomal pathway, with intraluminal vesicles being formed within multivesicular bodies and subsequently released upon their fusion with the plasma membrane. Exosomes are smaller than microvesicles, typically measuring between 30–150 nm in diameter. Apoptotic cells release a third type of EV named apoptotic bodies [8].

EVs are particularly rigid as their membranes are enriched in glycosphingolipids, cholesterol, phosphatidylserine, and ceramide [9]. Despite EVs released from different cell types express heterogeneous patterns of surface proteins, some recurrent molecular markers can be identified, including tetraspanins (CD9, CD63, CD81), Alix, HSP70, HSP90, GTPases (EEFs-1a1 and 2), and MHC molecules [9]. Furthermore, EVs also express cell-to-cell adhesion molecules, such as integrins and ICAMs, and several membrane receptors, such as scavenger receptors, complement receptors, EGFR, and TIM-4 [9,10,11]. The nucleic acid and protein cargo composition of EVs may reflect the pathophysiological state of the cells from which they derive [12,13]. Thus, with their stable detectability in body fluids and their minimally invasive sampling process, EVs have the potential to serve as clinically useful biomarkers, offering multiple insights into various disease statuses [13].

In LT, EVs have emerged as promising biomarkers to assess the risk of decompensation in cirrhosis, improve early diagnosis of hepatocellular carcinoma (HCC) before and after LT, refine the diagnosis of allograft rejection, and monitor the adequacy of immunosuppression [14,15]. Furthermore, mesenchymal stem cells (MSCs) and EVs have been shown to mitigate IRI in experimental models and can be synergically applied with MP [8,11,16,17,18].

To provide a snapshot of this rapidly evolving field, this review summarizes the most recent and promising applications of EVs as diagnostic and therapeutic tools in the setting of LT.

## 2. Results

### 2.1. EVs as Diagnostics Tools

#### 2.1.1. Liver Cirrhosis

Liver cirrhosis is an important cause of morbidity and mortality, accounting for over 2% of all global deaths. While HBV and HCV infections, despite eradication programs, remain the primary causes of chronic liver disease, in recent years, there has been a notable increase in dysmetabolic and alcohol-related cirrhosis [19]. LT in the setting of cirrhosis is considered in case of decompensation, complications of portal hypertension, or development of HCC. Plasma concentrations of EVs have been found to correlate with the severity of cirrhosis and can predict the risk of decompensation and mortality, as they play a crucial role in the pathogenesis and progression of liver injury [12]. Hepatocyte-derived EVs have shown a correlation with the degree of portal hypertension and the ability to predict 6-month mortality independently of Child–Pugh and Model for End-Stage Liver Disease scores, particularly when their plasma concentration exceeds 65 IU/mL [20]. Similarly, in patients with decompensated cirrhosis, low levels of EVs in ascites have been associated with a 30-day survival rate of 72%, compared to 95% in patients with high ascites–EV levels [21].

#### 2.1.2. Acute Liver Failure

In contrast to liver cirrhosis, acute liver failure (ALF) is becoming less frequent as an indication for LT, which nonetheless remains the only viable option for patients who do not benefit from medical management, representing approximately 30% of ALF cases [22]. One key issue in ALF is prognostic stratification to identify those patients who will ultimately require LT in a timely manner [23,24]. Stravitz et al. [25] found that a procoagulant phenotype, as well as specific size ranges of plasma EVs, correlate with complications and adverse outcomes in ALF patients. Authors reported that EVs ranging from 0.28 to 0.64 μm measured on days 1 and 3 from hospital admission were higher in patients who subsequently died or needed LT.

#### 2.1.3. Alcoholic Hepatitis

Another setting in which prognosis definition is essential is alcoholic hepatitis (AH) not responding to steroid therapy, an emerging—though much debated—indication for LT [26]. In AH patients, hematopoietic and hepatocyte-derived EV levels detected in peripheral blood prior to the initiation of steroid therapy correlated with the response to medical treatment and predicted 1- and 3-month survival [27].

#### 2.1.4. Liver Cancer

EVs as diagnostic tools have been widely investigated in the setting of liver cancer. HCC incidence is steadily increasing, making it the third leading cause of cancer-related death worldwide [28]. LT offers the highest survival benefit for HCC patients, but it is associated with a recurrence rate of 10–20% [29]. Serum biomarkers such as alpha-fetoprotein (AFP), des-gamma-carboxyprothrombin, and neutrophil-to-lymphocyte ratio are useful in diagnosing HCC and correlate with HCC recurrence after LT, even if there is no consensus on threshold values [30]. A primary objective of screening programs in cirrhosis is early HCC detection, and serum biomarkers play a crucial role. Several serum miRNAs have been reported as HCC biomarkers [31], and in recent years, the measurement of these miRNAs in serum EVs has raised considerable interest [32,33,34,35]. By actively regulating intercellular interaction, cell growth, and tissue invasion, EVs are detectable during the early stages of HCC [36]. Consequently, they could serve as valuable tools in the identification of patients requiring closer monitoring, facilitating the implementation of curative treatments, or potential consideration for LT [36,37].

Wang et al. reported that HCC patients have higher circulating EV values than cirrhotic patients without HCC [38]. The same authors found that circulating EVs were also related to HCC size, being more sensitive than AFP for early tumor detection [38]. Comparable results were recently reported by Xue et al., who observed that exosomal miRNAs were linked to the presence of HCC, and in particular, miRNA-106 correlated with HCC prognosis by actively stimulating cell proliferation [39]. Fang et al. [40] observed a positive correlation between increased serum levels of exosomal miRNA-1247-3p and the presence of lung metastases, which would exclude patients from LT. Additionally, the levels of miRNA-21- and lncRNA-ATB-containing EVs have been identified as independent predictors of overall survival and disease progression in HCC patients [41]. On this preliminary basis, a screening model based solely on serum biomarkers, replacing ultrasound scans, has been proposed, but this hypothesis still requires large-scale testing [42].

Recently, serum EVs have emerged as biomarkers capable of predicting HCC recurrence after LT. In patients with HCC recurrence after living donor LT (LDLT), miR-718 was significantly downregulated in circulating EVs [43]. Reduced miR-718 expression was associated with larger and poorly differentiated HCC recurrence due to the lack of inhibition of HOXB8, which suppresses cell proliferation. Nakano et al. [44] reported higher circulating exosomal levels of miR-92b both before and 1 month after LDLT in patients with HCC recurrence. Moreover, the combination of AFP and miR-92b was more accurate in predicting early recurrence.

Cholangiocarcinoma (CCA) is the second most frequent primary liver malignancy often associated with cholestatic diseases like primary sclerosing cholangitis (PSC) [45]. LT has become an established treatment option for unresectable perihilar CCA, following a stringent neoadjuvant chemoradiation regimen or in the case of a cirrhotic liver with a single intrahepatic nodule smaller than 3 cm [46,47]. EVs may improve diagnosis and risk stratification in CCA. A recent multicenter study observed that serum EV proteins can predict the development of CCA more than 1 year before clinical evidence of malignancy, allowing for curative treatment. Furthermore, specific EV profiles were associated with different CCA subtypes characterized by different prognoses [48]. Additionally, Li et al. [49] identified a panel of miRNAs derived from biliary EVs capable of distinguishing between malignant (CCA-related) and benign biliary stenosis, even in patients with PSC. Julich-Haertel et al. [50] observed increased levels of AnnexinV + EpCAM + ASGPR1 + EVs in patients with primary liver tumors (HCC or CCA) compared to cirrhotic patients without malignancy. It is worth noticing, however, that although EVs were identified as potentially useful biomarkers to improve early diagnosis, they were not specific for a particular type of liver neoplasm.

When LT is considered in the case of unresectable hepatic metastases from colorectal cancer, patient selection is crucial in order to achieve successful outcomes [51]. In this setting, levels of circulating tumor cells or circulating tumor-derived DNA can identify patients with residual metastatic cells in the bloodstream. There are currently no reported studies on the role of EVs in this context [52], and further research is warranted.

A schematic overview of the current utility of EVs for the diagnosis of liver diseases in the LT setting is provided in Table 1.

### 2.2. EVs and Rejection

#### 2.2.1. EVs and the Immune System

Rejection occurs when the immune system recognizes non-self antigens and activates against them. It can affect transplanted organs or tissues and involves various immunological components such as T lymphocytes (particularly CD8+ and CD4+ T cells), B lymphocytes, and cytokines.

Rejection can manifest in a hyperacute form within hours after transplantation when pre-existing antibodies against antigens are present, thus triggering a sudden and violent immune response with severe damage to the transplanted organ or tissue. When no antibodies are present in the recipient’s organism, acute rejection can occur through T-cell-mediated mechanisms, leading to inflammation and tissue damage. Finally, chronic rejection can be observed over years after transplantation. In this case, the molecular bases are poorly understood, and different phenomena—such as inflammation, fibrosis, endothelial cell injury, and antibody responses—are observed.

To mitigate transplant rejection, immunosuppressive agents are administered to suppress the recipient’s immune response against the transplanted liver. Nevertheless, T-cell-mediated rejection (TCMR)—which is the most frequent form and typically occurs within 3 months of transplantation—is observed in 10–30% of cases and is one of the most frequent early complications following organ transplantation [53].

When acute or chronic rejection is present, HLA molecules—also known as major histocompatibility complex (MHC) in humans—are among the main molecular actors, being responsible for antigen presentation and activation of the immune response against the graft.

**Figure 1:**
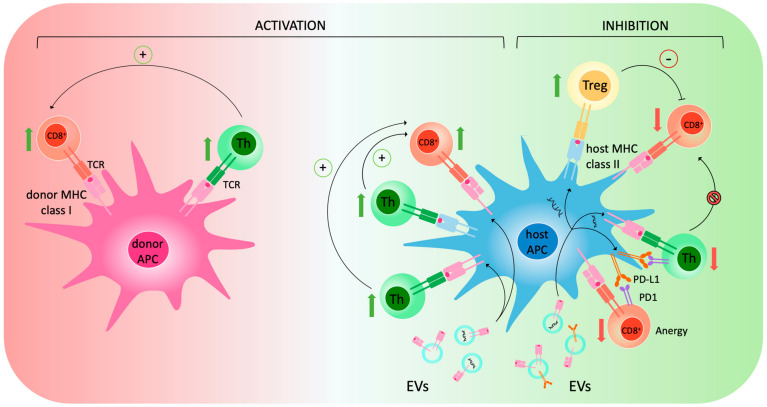
Schematic representation of the EV-dependent and independent mechanisms of immune activation and inhibition following LT. The direct mechanism involves T-cell activation by antigens exposed on donor APCs—also referred to as “passenger leukocytes”—while the indirect mechanism depends on T-cell activation by recipient APCs exposing processed donor antigens together with self-MHC. The semi-direct pathway involves the expression of EV-derived, intact donor MHC complexes on host APCs—a phenomenon also referred to as cross-dressing. Cross-dressed APCs can activate CD8+ T cells via MHC class I antigen presentation or CD4+ cells via MHC class II. These interactions generate a three-cell model which is responsible for immune activation. In the case of LT, EVs bearing PD-L1 on their surface induce the expression of such molecule on the host APC’s membrane, possibly due to molecule transfer and miRNA-dependent gene upregulation. The interaction between PD-L1 and PD1 determines anergy of CD8+ T cells and T-helper inhibition. Treg activation has also been reported as a possible mechanism of immune suppression. It has been proposed that such activation is EV-dependent and contributes to tolerance through the inhibition of CD8+ T cells. Abbreviations: antigen-presenting cell (APC), cytotoxic T lymphocytes (CD8+), major histocompatibility complex (MHC), programmed cell death protein 1 (PD1), programmed death ligand 1 (PD-L1), T-cell receptor (TCR), T-helper lymphocytes (Th), T regulatory lymphocyte (Treg).

In the context of transplantation, the induction of immune responses to MHC-mismatched allografts has been demonstrated to depend on two main pathways: the direct and indirect pathways [14,54]. The direct mechanism involves T-cell activation by antigens exposed on donor cells—also referred to as “passenger leukocytes”—and takes place in lymphoid organs, whereas the indirect mechanism depends on T-cell activation by recipient Antigen-presenting cells (APCs) exposing processed donor antigens together with self-MHC [55]. There is, however, a third possibility: the semi-direct pathway, which is defined by the expression of intact donor MHC complexes on recipient APCs—a phenomenon also referred to as cross-dressing [14,15]. Cross-dressed APCs can activate CD8+ T cells via MHC class I antigen presentation or CD4+ cells via MHC class II. These interactions generate a three-cell model that is responsible for immune activation and is schematically represented in Figure 1. The direct mechanism has long been thought to be predominant in acute rejection, while the indirect mechanisms might promote chronic rejection [14]. However, it has been recently observed that early after transplantation, very few passenger donor leukocytes are mobilized, while many recipient APCs carrying donor MHC molecules reach circulation and secondary lymphoid organs, thus taking part in immune modulation [56,57]. This evidence has questioned the exclusive role of the direct mechanism in acute rejection, bringing the semi-direct alloresponse to the attention of the scientific community. The first evidence of an exchange of MHC molecules between leukocytes dates back to 1974 [58], but it was not until the 2000s that this phenomenon was described in the context of transplantation [59]. In particular, the mechanism of cross-dressing was first studied in murine models of skin and heart transplantation, highlighting the prominent role of EVs in intercellular cross-talk [56,57]. Indeed, EVs can carry donor MHC and fuse with recipient lymphocytes, which then expose on their surface a graft-derived molecule capable of activating an immune response. Therefore, EVs can activate immune response both by mediating the cross-dressing of recipient leukocytes and—to a much lower extent—per se [14]. Further studies highlighted the role of EVs in hand [60] and islet [61] transplantation. In the context of LT, a paper by Mastoridis and colleagues [62] demonstrated that cross-dressed recipient leukocytes can be found in the circulation right after transplantation in much higher numbers than passenger leukocytes and that their levels decrease over time until becoming almost undetectable.

Besides their role as mediators of immune activation against the graft, evidence is rapidly growing on the opposite effect of EVs in selected settings such as feto-maternal tolerance [63,64], post-natal tolerance of non-inherited maternal antigens [65], intestine [66], and even LT [67], where EVs have been shown to exert an immunoinhibitory effect. The molecular mechanisms underlying immune inhibition are multiple and not fully understood, but it has been proposed that EVs induce Treg activation and CD4+ and CD8+ lymphocytes anergy [14]. Other factors that may explain why EVs can sometimes induce immune inhibition rather than activation are their amount, their surface phenotype and molecular cargo, as well as the site of generation/interaction. In the case of LT, in fact, EVs are generated in a microenvironment in which PD-L1 is highly upregulated [67,68]. This favors the generation of EVs bearing PD-L1 on their surface, which activates the PD1/PD-L1 axis on immune cells generating lymphocytes anergy and tolerance. Of note, it has been observed that EVs generated from other organs, such as the kidney, do not show the same immunomodulatory properties as those generated in transplanted livers [62], which may favor tolerance. A schematic representation of the mechanisms through which EVs may induce immune inhibition is shown in Figure 1.

Based on what has been described so far, EVs and their role in cross-dressing appear as important actors in the development of rejection. Thus, interfering with such dynamics might be a valuable option to dampen the immune response and ameliorate allograft survival.

Although little to no evidence is now available in a human setting of solid organ transplantation, it has been proposed that EV-driven CD47 overexpression in APCs could prevent phagocytosis [69], thus mitigating allo-directed immune response. In such a context, it can be speculated that genetic modifications hampering cross-dressing—similar to the one described—may be a promising novel approach in the treatment of rejection.

#### 2.2.2. Applications of EVs in Rejection

Besides being involved in immune regulation, EVs can also be exploited as potential biomarkers to help with the diagnosis of acute rejection, as their cargo seems dependent on the presence of liver damage. Zhang and colleagues showed that EVs derived from blood samples of patients presenting with acute rejection after LT are particularly rich in galectin-9, as are the cells from the selected livers of origin [70]. This observation may be helpful in designing a non-invasive way to refine rejection diagnosis and monitor response to treatment in transplanted patients. Similarly, Wang et al. demonstrated that miR-223, let-7e-5p, miR-486-3p, miR-199a-3p, miR-148a-3p, and miR152-3p are selectively modulated in EVs isolated from the sera of transplanted patients with acute rejection as compared to control patients [71], suggesting their potential role as diagnostic biomarkers.

Recent evidence on the relationships between EVs and rejection has prompted the possibility of using them as a therapy in patients presenting with rejection. In parallel with graft-derived EVs, other types of vesicles have been studied. Among these, mesenchymal stem-cell-derived EVs (MSC-EVs) have been shown to be able to transfer specific molecules to the recipient immune cells. Zhou et al. recently demonstrated that MSCs-EVs can carry specific miRNA (miR-22-3p) into Kupffer cells, inhibiting IRF8 expression and inducing M2 polarization [72]. This results in local immune suppression and downmodulation of liver rejection.

A recent paper also reported that CD80+ dendritic cell-derived EVs could also be a good therapeutic candidate. The authors observed a decrease in CD80+ dendritic cells in the liver of patients with acute rejection while a rich infiltrate of CD8+ T and a high expression of NLRP3 and Ki67 were present. T cells exposed to CD80+ dendritic cell-derived EVs downregulated NLRP3 expression and showed reduced proliferation, adhesion, and invasion, suggesting induced tolerance [73].

### 2.3. Therapeutic Applications of EVs

Stem cell therapy has enormous potential in the treatment of liver diseases. MSCs have garnered significant attention due to their unique characteristics and therapeutic possibilities [6]. In addition to their ability to differentiate into multiple cell types, MSCs also have potent anti-inflammatory and immunomodulatory effects that were already demonstrated in experimental models of LT [8]. However, the therapeutic effects of stem cells primarily result from the release of various paracrine factors, with EVs serving as key mediators. In contrast to stem cell therapies, EVs offer several advantages, such as low immunogenicity, non-tumorigenicity, ease of storage, and high clinical safety, while retaining equivalent therapeutic properties [10,74].

#### 2.3.1. Mechanisms of Protection against Liver IRI

In the liver, EVs’ protective mechanisms against IRI point mainly towards immune response modulation [75,76,77,78,79,80], autophagy regulation [81,82], and activation of regenerative pathways [83,84,85]. The most commonly utilized murine model of IRI involves the selective clamping of the hepatic pedicle, resulting in approximately 70% warm ischemia, followed by subsequent reperfusion, and this protocol has been applied to investigate the protective effects of EVs in all the following studies. In rat livers exposed to IRI, intravenous administration of MSC-EVs decreased neutrophil and macrophage infiltration, as well as oxidative stress markers [75,76]. Similarly, Haga et al. [77] found that MSC-EVs decreased liver IRI by reducing the secretion of pro-inflammatory cytokines, such as TNF-α, IL-1α, IL-1β, IL-6, IL-12, and IFNγ. In a study by Sun et al. [78], MSC-EVs administration in rats resulted in decreased liver injury and immune cell infiltration, accompanied by lower release of pro-inflammatory cytokines, activation of mitochondrial damage, and oxidative stress. Notably, the combination of MSC-EVs and melatonin resulted in the highest level of protective effects across all study endpoints. Two other groups recently identified ERK1/2 activation and GSK-3β inactivation [79], as well as the inhibition of MAPK and NF-κB pathways [80], respectively, as the possible molecular mechanisms underlying the hepatoprotective effects of MSC-EVs in liver IRI.

Yang et al. [81] differentiated mouse bone-marrow-derived MSCs into an MSC-hepatocyte transitional phenotype cell and isolated EVs from these cultured cells. In this protocol, mice were injected twice with such EVs before hepatic clamping and at reperfusion, resulting in lower markers of liver IRI and restored autophagic activity, as confirmed by increased Beclin-1 and LC3-II expression. By contrast, in vivo injection of MSC-EVs, particularly after cotreatment with microRNA-20a, reduced aberrant expression of autophagy mediators LC3-II and Beclin-1, resulting in higher protection against IRI [82]. These findings confirmed the central role of autophagy in liver IRI and highlighted the need for further elucidation of its mechanism to be targeted with EVs.

Du et al. [83] investigated the effects of MSC-EVs on hepatocyte proliferation after IRI in rats and found that EV injection induced hepatocyte proliferation by activating the sphingosine kinase/sphingosine 1-phosphate pathway. Accelerated proliferation was also detected by Ki67 staining in mouse livers treated with MSC-EVs prior to IRI [84]. Notably, while all other pro-inflammatory markers were downregulated, IL-6 was postulated to be the effector of liver regeneration through the activation of the STAT3 pathway. Interestingly, IL-6-mediated regeneration was also recently observed in hepatectomized rat livers injected with MSC-EVs [85].

Human liver stem-like cells (HLSCs) have been identified as a pluripotent population of liver resident cells expressing markers characteristic of both the mesenchymal and hepatic lineage, indicating partial hepatic commitment [86]. HLSCs and their extracellular vesicles (HLSC-EVs) have been shown to bear protective effects, such as anti-inflammatory, anti-apoptotic, and pro-regenerative properties, that make them promising candidates for cell-based therapies in liver diseases [87,88,89]. Similarly to MSC-EVs, we recently demonstrated that HLSC-EVs could ameliorate tissue injury in mouse livers exposed to IRI by downregulating TNF-α, CCL-2, and CXCL-10 expression [90].

#### 2.3.2. EV-Based Therapies during MP

One interesting application of EV-based therapies in LT would be their administration during MP. MP has brought a paradigm shift in organ preservation and its use, besides being associated with reduced IRI [91,92,93,94,95,96,97,98,99], has allowed safely extending preservation time [100,101] and testing liver viability before transplantation [102,103,104,105,106,107,108]. Furthermore, MP is being increasingly investigated as a platform to allow interventions aiming at improving donor quality, including the possibility of administering stem cells or EVs during perfusion. By establishing an isolated ex situ platform in which the organ is metabolically active, therapies targeting IRI can be delivered directly to the liver, limiting systemic exposure to the recipient [8]. Indeed, the effectiveness of delivering MSCs during perfusion to mitigate IRI has been demonstrated across multiple organ systems [109,110,111]. As previously mentioned, the utilization of EVs instead of stem cells presents various potential benefits that are further amplified by the implementation of MP. These advantages include the capability to traverse biological barriers, specificity in targeting, avoidance of obstruction in microvascular beds or circuit components, stability during storage, decreased possibility for phenotypic variation upon administration, comparatively lesser immunogenicity and tumorigenicity, and enhanced safety profiles during repeated administrations [8]. The combination of MP and EVs has already been successfully applied during liver, kidney, and lung perfusion (Table 2) [112,113,114,115,116,117,118,119,120]. In particular, our group has been the first to deliver HLSC-EVs using normothermic machine perfusion (NMP) of the liver [117]. After 4 h of hypoxic-NMP, the hepatocyte uptake of HLSC-EVs was confirmed by epifluorescence microscopy, and the treated livers showed reduced cytolysis and tissue injury, as well as overexpression of HIF-1α and TGF-β1. To further investigate HLSC-EVs effectiveness in a clinically relevant scenario, we performed an NMP model with prolonged warm ischemia time and found that HSLC-EVs-treated organs showed less transaminases release and better-preserved liver function, with enhanced pH self-regulation and phosphate utilization [118]. Remarkably, a further improvement was observed in bile production, hemodynamics, tissue necrosis, and cell proliferation when higher doses of HLSC-EVs were added to the perfusate, suggesting a dose–response correlation.

Although the preliminary studies on EV-based therapies during liver NMP are promising, more extensive research is necessary to optimize dosing strategies, evaluate the safety of repeated dosing, and investigate the translatability and efficacy of these protocols in vivo.

## 3. Discussion

**Figure 2:**
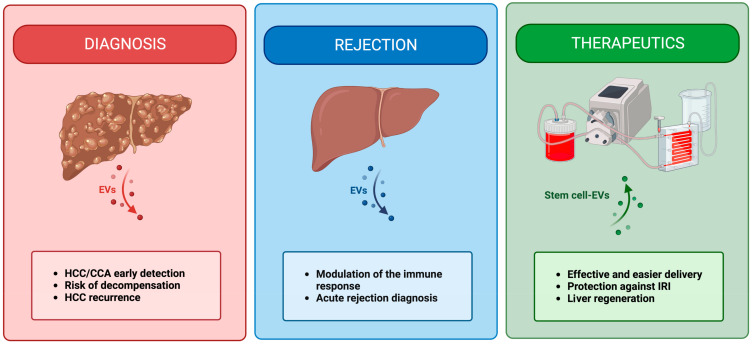
Schematic representation of potential applications of EVs in the LT setting. EVs show significant potential in LT, serving as valuable biomarkers for assessing liver disease severity and predicting adverse outcomes. They hold promise in monitoring patients, selecting suitable candidates for LT, and facilitating early detection of HCC. Moreover, EVs play a complex role in rejection, modulating immune responses, and offering diagnostic potential. Integration of EVs with MP appeared to be a valuable option to reduce IRI but requires standardization and regulatory guidelines for clinical use.

EVs have demonstrated significant potential in the field of LT and liver diseases (Figure 2).

Their plasma levels and molecular profiles can correlate with the severity of liver cirrhosis, providing an estimation of the risk of decompensation and mortality. Additionally, they hold promise in predicting adverse clinical outcomes in acute scenarios such as ALF and AH. These findings underscore the potential of EVs in monitoring patients and selecting appropriate candidates for LT. Moreover, EVs have emerged as valuable diagnostic tools in HCC, facilitating early detection and prognosis stratification. Hence, their use as biomarkers has raised considerable interest. However, further studies are necessary to evaluate their cost-effectiveness, establish diagnostic cut-offs, and assess their clinical utility in larger cohorts.

The role played by EVs in the context of rejection is complex, and many factors can influence the outcome of EV-immune cell interaction. The amount, surface phenotype, cargo, as well as origin cell, and generation/interaction site can, in fact, dramatically change their activity. In the context of LT, EVs can activate alloresponse by allowing recipient APCs to expose donor MHC complexes on their surface and activate T lymphocytes. On the other hand, in what seems to be a liver-specific mechanism, EVs can induce immune suppression through (i) cross-dressed APC-mediated recruitment and activation of Treg cells, (ii) PD1-PD-L1-mediated inhibition of T helper lymphocytes, and (iii) PD1-PD-L1-mediated induction of CD8+ lymphocytes anergy. Besides modulating the activity of the immune system against the graft, EVs can be used as efficient and non-invasive biomarkers, as their cargo seems to selectively change when rejection occurs. In this scenario, both miRNAs and proteins—selectively up- or down-modulated in circulating donor liver-derived EVs—may have a diagnostic potential.

The simultaneous advancement of MP and cell-based therapies has paved the way for the integration of these technologies. In addition to mitigating IRI and enhancing LT outcomes, MP provides a critical time frame for the administration of organ-specific treatments. Incorporating cell-based therapies during ex situ perfusion offers multiple benefits, such as the ability to optimize dosing and circumvent the drawbacks associated with systemic administration. In this sense, EVs provide significant biosafety advantages over standard stem cell therapies.

### Challenges to Clinical Translation

Despite promising preliminary results, challenges remain in the clinical translation of EVs. Several ongoing clinical trials primarily focus on assessing the safety and tolerability of EV-based treatments for cancer or SARS-CoV-2-related diseases [121]. Currently, only one trial is investigating MSC-EVs as a potential therapy for solid organ transplantation rejection [122] as clinical translation of EV-based therapies faces challenges due to the lack of quality control and standardization procedures [121].

Different laboratories employ different isolation and purification methods, leading to a lack of uniform protocols. Similarly, an accurate quantification of EVs is crucial when utilizing them as therapeutic agents, and various approaches based on particle number, dimensions, and total protein, lipid, or nucleic acid content are employed [123]. Each method has its limitations and unique challenges, which makes it difficult to determine the superiority of one approach over another.

Low yield also presents a barrier to the clinical translation of EVs, particularly in cases where EVs are derived from stem cells. While abundant cellular sources of EVs may not encounter this issue, stem-cell-based protocols may face technical and cost-related challenges. Researchers are actively exploring cell expansion strategies, culture conditions, and bioreactors to enhance EV production [121]. However, the dosage and method of EV administration remain a significant challenge. In a recent systematic review of EV-based therapies for transplantable organs, Blondeel et al. [124] reported a range of administered EV doses ranging from 10^5^ to 10^12^ particles, as well as an extreme variability in administration routes, including intravenous, intra-arterial, and intra-organ injection. This extreme methodological heterogeneity currently makes it impossible to conduct meaningful meta-analyses and a feasible scaling up to large-size organ models. On the other hand, as previously mentioned, the unique drug delivery platform offered by MP may serve as a potential solution to overcome these issues and optimize the translational process [8].

Following large-scale purification, maintaining appropriate storage conditions becomes essential to ensure the stability of EVs. The prevailing method involves resuspending EVs in phosphate-buffered saline and then storing them at −80 °C for up to 6 months [90,118]. This approach further increases the costs of the process and significantly complicates transportation logistics, particularly from the perspective of clinical translation. As a result, researchers are actively exploring alternative storage methods, but solid data are still lacking [121,123].

Safety is of utmost importance when considering EVs for clinical use. Immunogenicity, immunotoxicity, and potential carcinogenicity have been mentioned as safety concerns [121,125]. Balancing the potential benefits and risks, especially in transplanted patients undergoing immunosuppression, requires careful consideration.

Nevertheless, significant strides have been made in an attempt to improve the standardization and reproducibility of studies involving EVs, and rigorous guidelines with useful tools are already available [126].

## 4. Materials and Methods

The Medline (PubMed) database was accessed on 12 March 2023, using the search terms ‘extracellular vesicles’ AND ‘liver transplantation’ without any time limitations. Inclusion criteria comprised clinical and preclinical studies published in peer-reviewed journals focusing on the use of EVs in LT and/or their applications for diagnosis and treatment of LT-related diseases. There were no restrictions based on species, age, or sex. Case reports, letters to the editor, publications without full-text availability, and studies published in languages other than English were excluded. A total of 288 articles were retrieved. Three authors (NDS, AC, and ACF) performed the literature review, and any disagreements were resolved through consensus. Initially, 102 articles were selected for potential relevance by title screening. Subsequently, the abstracts of the selected articles were screened according to the inclusion and exclusion criteria, resulting in 64 articles eligible for full-text review. Additionally, 57 articles were identified through manual cross-checking of the cited references, and 5 more were included following peer review. In total, 126 articles were included in this narrative review.

## 5. Conclusions

EVs show substantial potential in LT by serving as valuable biomarkers for disease severity assessment and enabling early HCC detection. EVs play a multifaceted role in organ rejection and immune modulation, and its further exploration could pave the way for diagnostic and therapeutic advancements. The integration of MP technology with EVs holds significant promise in mitigating IRI, potentially addressing organ shortage by enabling the regeneration of organs that are currently being discarded.

However, standardizing EV separation and purification techniques, establishing quantitative standards, improving EV yield while maintaining homogeneity, ensuring proper storage, and rigorously assessing safety are all critical steps before the clinical translation of EV-based therapies in the transplant setting, considering the costly nature of their implementation within an already expensive procedure. Nevertheless, future research efforts will likely contribute to harnessing the full potential of EVs in LT, ultimately improving patient outcomes and shaping the future of personalized medicine.

## Figures and Tables

**Table 1:** Schematic overview of EVs as diagnostic tools for liver diseases in the LT setting.

Author	Disease	Population	EVs Subtype	Outcomes
Stravitz et al., 2013 [25]	ALF	50 ALI (39 ALF): 27 spontaneous survivors, 23 LT or death	- Platelet-derived EVs (CD41+) - Hepatocyte-derived EVs (ASGPR+)	Day 1 EV levels predicted the risk of LT or death Day 1 and 3 (0.28–0.64 µm) EVs were higher in patients who died or underwent LT
Wang et al., 2013 [38]	HCC	55 HCC 40 LC	NA	Blood EV levels were significantly higher in HCC patients compared to LC EV levels correlate with HCC stage EVs (cut-off 5.41 mcg/mL) had better sensitivity and specificity than AFP (cut-off 20 ng/mL) in early HCC detection
Sugimachi et al., 2015 [43]	HCC	65 LDLT for HCC	TSG101+	Exosomal miR-718 was downregulated in patients who presented HCC recurrence
Engelmann et al., 2017 [21]	aCLD	163 LC	- Absolute ascites EV levels - Neutrophils–EVs (CD66b+) - Lymphocytes–EVs (CD3+)	Low ascites EV levels (<488 EVs/µL) associated with reduced 30-day survival rate Higher amount of EVs derived from neutrophils and lymphocytes associated with reduced survival
Julich-Haertel et al., 2017 [50]	HCC/CCA	22 HCC 26 CCA	AnnexinV + EpCAM + tumor-associated EVs	EV levels allowed the distinction of liver malignancies (HCC or CCA) and tumor-free cirrhosis EV levels correlated moderately with liver tumor diameter
Payancè et al., 2018 [49]	aCLD	242 LC	Hepatocyte-derived EV (CK-18+)	Hepatocyte EV levels correlate weakly with HVPG Hepatocyte EVs > 65 U/L predict 6-month mortality
Sukriti et al., 2018 [27]	AH	101 AH (71 responders, 30 non-responders to steroid therapy)	- Hematopoietic stem cells (CD45 + CD34+) - Hepatocytes (ASGPR+)	Baseline serum EV levels predicted steroid non-response in 94% of cases
Xue et al., 2019 [39]	AH	80 HCC 30 healthy controls	- CD9^+^–EVs - CD63^+^–EVs	Exosomal miR-106a was a prognostic factor for HCC, predicting 2- and 10-year survival
Fang et al., 2018 [40]	HCC	90 HCC without lung metastasis 20 HCC with lung metastasis	HCC-derived EVs	High miR-1247-3p expression was well predicted for poor OS and poor DFS High serum exosomal miR-1247-3p expression is correlated with lung metastasis
Lee et al., 2019 [41]	HCC	79 HCC	- CD9^+^–EVs - CD63^+^–EVs - TSG101^+^–EVs	miRNA-21 and lncRNA-ATB were related to TNM stage and PVT OS and PFS were lower in patients with higher values of exosomal miRNA-21 and lncRNA-ATB
Nakano et al., 2019 [44]	HCC	93 HCC pts who underwent LDLT	NA	Increase in exosomal miR-92b before LDLT reflects a risk for posttransplant early HCC recurrence
Sorop et al., 2020 [35]	HCC	48 HCC 38 LC	- CD63^+^–EVs - CD9^+^–EVs - CD81^+^–EVs	Exosomal miR-21-5p was upregulated, and miR-92a-3p was downregulated in HCC patients
Lapitz et al., 2023 [48]	CCA	45 PSC 69 PSC-CCA 56 CA 34 HCC	- CD63^+^–EVs - CD81^+^–EVs	Serum EV proteins allowed the prediction of CCA development in patients with PSC before clinical evidence of malignancy Serum EVs aid the differential diagnosis between HCC and iCCA

Abbreviations: advanced chronic liver disease (aCLD), alpha-fetoprotein (AFP), alcoholic hepatitis (AH), acute liver failure (ALF), acute liver injury (ALI), cholangiocarcinoma (CCA), disease-free survival (DFS), extracellular vesicles (EVs), hepatocellular carcinoma (HCC), hepatic venous pressure gradient (HVPG), liver cirrhosis (LC), liver transplantation (LT), living donor LT (LDLT), microRNA (miRNA), not assessed (NA), non-coding RNA (ncRNA), progression-free survival (PFS), primary sclerosing cholangitis (PSC), portal vein thrombosis (PVT).

**Table 2:** Schematic overview of experimental studies combining EVs and MP to mitigate IRI.

Author	Organ	Injury	Perfusion	Timing	Treatment	Dose	Outcomes
Gennai et al., 2015 [112]	Human lungs	Grafts rejected for transplant	Normothermic	8 h	BM-MSC-EVs	100 or 200 μL of supernatant (10 μL isolated from 1 × 10^6^ cells)	↑ Alveolar fluid clearance, ↑ Pulmonary compliance ↓ PAP and PVR
Stone et al., 2017 [113]	Mouse lungs	Warm ischemia (60 min)	Normothermic	1 h	Umbilical cord derived-MSC-EVs	1 × 10^6^ EVs prior to ischemia and 3 × 10^6^ EVs during perfusion	↑ Pulmonary compliance ↓ PAP ↓ Edema and neutrophil infiltration
Gregorini et al., 2017 [116]	Rat kidney	Warm ischemia (20 min)	Hypothermic	4 h	BM-MSC-EVs	EVs isolated from 3 × 10^6^ cells	↓ Tissue injury ↓ Lactate, LDH, MDA
Rigo et al., 2018 [117]	Rat liver	Hypoxic injury	Normothermic	4 h	HLSC-EVs	5 × 10^8^ EVs/g liver	↓ AST, LDH ↓ tissue injury, apoptotic cells ↓ HIF-1α, TGF-β1
Park et al., 2019 [114]	Human lungs	Grafts rejected for transplant with *E. coli* pneumonia	Normothermic	6 h	BM-MSC-EVs	200 or 400 μL of supernatant (10 μL × 1 × 10^6^ cells)	↑ Alveolar fluid clearance
Lonati et al., 2019 [115]	Rat lungs	-	Normothermic	3 h	MSC-EVs	24.56 ± 5.53 × 10^10^ EVs	↓ PVR ↑ NO metabolites and ATP
De Stefano et al., 2021 [118]	Rat liver	Warm ischemia (60 min)	Normothermic	6 h	HLSC-EVs	5 × 10^8^ EVs/g liver 25 × 10^8^ EVs/g liver	↓ AST, ALT, phosphates, ↓ Total HCO_3_^−^ need ↑ Bile production (High dose only) ↓ Necrosis ↑ proliferation (High dose only) ↓ Vascular resistance (High dose only)
Rampino et al., 2022 [119]	Human kidney	Grafts rejected for transplant	Hypothermic	4 h	BM-MSC-EVs	28.5 × 10^9^ EVs	↓ Tissue injury ↓ caspase-3 ↑ COX IV-1, HGF and VEGF
Grignano et al., 2022 [120]	Rat kidney	Warm ischemia (20 min)	Hypothermic	4 h	BM-MSC-EVs or BM-MSC-EVs silenced for CD73	EVs isolated from 3 × 10^6^ cells	↓ Tissue injury ↑ ATP and tubular proliferation Silencing CD73 abolished protection

Abbreviations: alanine aminotransferase (ALT), aspartate aminotransferase (AST), adenosine triphosphate (ATP), bone marrow mesenchymal stem-cell-derived extracellular vesicles (BM-MSC-EVs), cytochrome c oxidase IV-1 (COX IV-1), hepatocyte growth factor (HGF), human liver stem-cell-derived extracellular vesicles (HLSC-EVs), hypoxia-inducible factor 1α (HIF-1α), lactate dehydrogenase (LDH), malondialdehyde (MDA), nitric oxide (NO), pulmonary arterial pressure (PAP), pulmonary vascular resistance (PVR), transforming growth factor- β1 (TGF-β1), vascular endothelial growth factor (VEGF), ↑ increase, ↓ decrease.

## Data Availability

No new data were created or analyzed in this study. Data sharing is not applicable to this article.
